# Real-Time Monitoring of Key Gene Products Involved in Rice Photoperiodic Flowering

**DOI:** 10.3389/fpls.2021.766450

**Published:** 2021-12-15

**Authors:** Hayato Yoshioka, Keiko Kimura, Yuko Ogo, Namie Ohtsuki, Ayako Nishizawa-Yokoi, Hironori Itoh, Seiichi Toki, Takeshi Izawa

**Affiliations:** ^1^Laboratory of Plant Breeding and Genetics, Department of Agricultural and Environmental Biology, Graduate School of Agricultural and Life Sciences, The University of Tokyo, Tokyo, Japan; ^2^Institute of Crop Science, National Agriculture and Food Research Organization, Tsukuba, Japan; ^3^Institute of Agrobiological Sciences, National Agriculture and Food Research Organization (NARO), Tsukuba, Japan; ^4^Graduate School of Nanobioscience, Yokohama City University, Yokohama, Japan

**Keywords:** rice, photoperiodic flowering, short-day plant, luciferase (LUC), circadian clock

## Abstract

Flowering is an important biological process through which plants determine the timing of reproduction. In rice, florigen mRNA is induced more strongly when the day length is shorter than the critical day length through recognition of 30-min differences in the photoperiod. *Grain number, plant height, and heading date 7 (Ghd7)*, which encodes a CCT-domain protein unique to monocots, has been identified as a key floral repressor in rice, and *Heading date 1 (Hd1)*, a rice ortholog of the *Arabidopsis* floral activator *CONSTANS (CO)*, is another key floral regulator gene. The Hd1 gene product has been shown to interact with the Ghd7 gene product to form a strong floral repressor complex under long-day conditions. However, the mRNA dynamics of these genes cannot explain the day-length responses of their downstream genes. Thus, a real-time monitoring system of these key gene products is needed to elucidate the molecular mechanisms underlying accurate photoperiod recognition in rice. Here, we developed a monitoring system using luciferase (LUC) fusion protein lines derived from the *Ghd7-LUC* and *Hd1-LUC* genes. We successfully obtained a functionally complemented gene-targeted line for *Ghd7-LUC*. Using this system, we found that the Ghd7-LUC protein begins to accumulate rapidly after dawn and reaches its peak more rapidly under a short-day condition than under a long-day condition. Our system provides a powerful tool for revealing the accurate time-keeping regulation system incorporating these key gene products involved in rice photoperiodic flowering.

## Introduction

The floral transition is an important biological event in which a plant switches from the vegetative phase to the reproductive phase. Many plants monitor changes in external environmental factors such as temperature and the photoperiod to release offspring during the most favorable season. Because plants recognize the photoperiod using their leaves, whereas meristems at the shoot apex form flower buds, the existence of a hormone-like substance that is transferred from the leaves to meristem has been hypothesized, with this substance described as “florigen” (Chailakhyan, 1937). Based on extensive molecular genetic analysis of floral formation, florigen has been identified as evolutionarily conserved small proteins encoded by genes such as *FLOWERING LOCUS T* (*FT)* (Kardailsky et al., 1999; Kobayashi et al., 1999) in *Arabidopsis thaliana* and two orthologous genes in rice, *Heading date 3a* (*Hd3a*) and *RICE FLOWERING LOCUS T* 1 (*RFT1*) (Kojima et al., 2002; Corbesier et al., 2007; Tamaki et al., 2007).

In *A. thaliana*, a long-day plant, the zinc-finger transcription factor *CONSTANS (CO)* directly induces *FT* gene expression in a photoperiod-dependent manner (Samach et al., 2000). *CO* mRNA expression is regulated by the circadian clock gene *GIGANTIA* (*GI*) (Suárez-López et al., 2001). The *GI-CO-FT* pathway is currently recognized as an evolutionarily conserved genetic pathway that controls flowering timing in many plants.

In rice, a short-day plant, the *Hd3a* gene is induced under short-day conditions when a critical day-length threshold (approximately 13.5 h) is recognized, in contrast to *A. thaliana* (Itoh et al., 2010). Rice *Hd3a* and *RFT1* is regulated mainly through two genetic pathways, one of which corresponds to the *GI-CO-FT* pathway in *A. thaliana*. The rice circadian clock, in which the *OsGIGANTEA (OsGI)* gene (Hayama et al., 2002; Izawa et al., 2011) is a major component, controls the *Heading date1 (Hd1)* gene, a rice ortholog of *CO*, and *Hd1* in turn regulates *Hd3a* and *RFT1* (Hayama et al., 2003). Thus, the *OsGI-Hd1-Hd3a/RFT1* pathway is orthologous to the *GI-CO-FT* genetic pathway in *A. thaliana*. Notably, *CO* promotes *FT* expression under long-day conditions in *A. thaliana*, whereas *Hd1* promotes *Hd3a* and *RFT1* expression under short-day conditions and represses those genes under long-day conditions in rice (Izawa et al., 2002). The other genetic flowering pathway is unique to monocotyledonous plants. Both the *Early heading date1 (Ehd1)* gene (Doi et al., 2004; Izawa, 2007), which encodes a B-type response regulator and mainly induces *Hd3a* and *RFT1* expression under short-day conditions, and the *Grain number, plant height and heading date 7* (*Ghd7*) gene, which encodes a CO, CO-like, and TOC1 (CCT)-domain protein (Xue et al., 2008) that functions as a strong floral repressor, were identified as flowering-timing genes unique to monocotyledonous plants. *Ghd7* directly suppresses the transcription of *Ehd1*, which in turn promotes the transcription of *Hd3a* and *RFT1* (Itoh et al., 2010; Nemoto et al., 2016). Thus, the *Ghd7-Ehd1-Hd3a/RFT1* pathway exists in rice in addition to the evolutionarily conserved *OsGI-Hd1-Hd3a/RFT1* pathway. In addition, it is of note that *OsGI* can contorl *Ehd1* as a part of the blue light signaling pathway (Itoh et al., 2010; Izawa et al., 2011). Several years ago, we reported a molecular genetic interaction between these two pathways. *Hd1* alone promotes *Ehd1* at night under short-day conditions, whereas *Hd1* and *Ghd7* cooperate to suppress *Ehd1* transcription during daytime under long-day conditions through the formation of a floral suppressor complex including both Ghd7 and Hd1 gene products, which can bind to the promoter region of *Ehd1* (Nemoto et al., 2016). This finding partially explains the molecular mechanism through which *Hd1* promotes flowering under short-day conditions and suppresses it under long-day conditions, although *Ghd7* exhibits significant repressor activity even in a background with *hd1* deficiency.

These findings demonstrate that the diurnal dynamics of *Hd1* and *Ghd7* mRNA cannot explain how rice precisely recognizes day length and controls downstream genes such as *Hd3a/RFT1* in leaves (Nemoto et al., 2016). Thus, a monitoring system for the Hd1 and Ghd7 gene products, such as a luciferase (LUC) reporter gene fused to the target gene in an in-frame manner, is needed.

Among higher plants, gene-targeting (GT) technology has not yet been put to practical use because in plant somatic cells, the main repair mechanism for double strand breaks (DSBs) is non-homologous end joining, rather than homologous recombination (HR) (Salomon and Puchta, 1998). In the 1990s and early 2000s, studies on GT involving the introduction of homologous sequences into cells of higher plants such as tobacco, *A. thaliana*, and rice were reported, but the incidence of GT ranged from 1/10^4^ to 1/10^5^ per transformation event, which was very low (Puchta and Fauser, 2013). In 2002, Terada et al. introduced a positive and negative selection method for GT in rice. This method was relatively efficient for GT in higher plants. A few years ago, a method using CRISPR/Cas9 technology was reported in *A. thaliana*, and verification of its versatility has just begun (Miki et al., 2018). In addition, CRISPR/Cas9 mediated GT system was applied for rice and tobacco (Nishizawa-Yokoi et al., 2020). Because the regulatory region around the target gene is completely intact in such GT reporter lines, the original dynamic pattern of the target protein can be accurately monitored *in vivo.*

**FIGURE 1:**
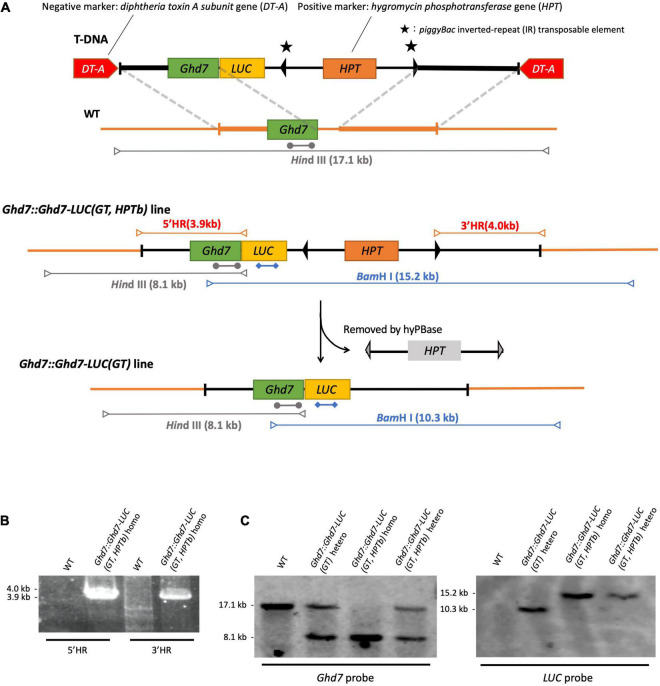
Gene-targeting of *LUC* gene fused to *Ghd7* gene. **(A)** Outline of experimental strategy of gene targeting. *Hin*dIII-cut fragments (gray arrows), *Bam*HI-cut fragments (blue arrows), *Ghd7* probe (gray bars; 1273 kb), LUC probe (blue bars; 974 bp), and PCR fragments (red arrows). The gene-targeted *Ghd7::Ghd7-LUC (GT,HPTb)* line was properly selected by PCR screening **(B)** and by Southern blot hybridization **(C)**. Primer sequences are listed in Supplementary Table 1.

In this study, we adopted a GT system using positive and negative selection (Nishizawa-Yokoi et al., 2015; Shimatani et al., 2015; Figure 1A). Then, we successfully constructed monitoring systems using a gene-targeted (GT) Ghd7-LUC and random-integrated (RI) Hd1-LUC proteins. Using those lines, we were able to observe dynamic patterns of Ghd7 and Hd1 protein levels *in vivo* under various environmental and genetic conditions to reveal the dynamic regulation between upstream Hd1 and Ghd7 gene products and downstream transcripts of the *Ehd1*, *Hd3a*, and *RFT1* genes. The general temporal dynamics of the Ghd7-LUC and Hd1-LUC proteins under both short-day and long-day conditions are reported in this work.

## Materials and Methods

### Vector Construction for Gene Targeting

Two *diphtheria toxin A fragment* (*DT-A*) genes under the control of the maize polyubiquitin 1 promoter (Pubi) or rice elongation factor-1α promoter (Pef) (Terada et al., 2002) within the right and left border sequences of the T-DNA vector were used as negative selection marker genes in this study (Nishizawa-Yokoi et al., 2015), whereas the *hygromycin phosphotransferase* (*HPT*) gene containing *piggyBac* (Cary et al., 1989) inverted repeat (IR) sequences as flanking sequences was used as a positive selection marker. The *piggyBac* IR sequences can be used to excise the *HPT* gene after GT using the transformed hyperactive *Piggybac* transposase [*hyPBase* (Yusa et al., 2011)]. Five polymerase chain reaction (PCR) fragments were designed and designated F1–F5 (Supplementary Figures 1, 2) as follows: F1, promoter region and coding sequence region of the target gene; F2, LUC coding sequence; F3, 3′ untranslated region sequence of the target gene; F4, the positive selection marker (*HPT* gene); and F5, the subsequent 3′ region of the target gene. These fragments were fused *via* the SLiCE (seamless ligation cloning extract) (Okegawa and Motohashi, 2015) method or using an In-Fusion HD cloning kit (Takara Bio United States, Inc.) and sub-cloned into the T-DNA vector containing two *DT-A* negative selection marker genes. The fragments F1 and F2 were designed to produce the target gene product fused to the *LUC* gene in-frame, whereas fragments F3, F4, and F5 were designed to reproduce the intact 3′ region sequences after the excision of *piggyBac*. The sub-cloned sequences were verified using the conventional Sanger sequencing method. In this work, the target genes were *Ghd7* and *Hd1* in rice (Supplementary Figures 1, 2). Finally, we constructed the pKOD4/Hd1::Hd1-LUC pHPTb and pKOD4/Ghd7::Ghd7-LUC pHPTb vectors (Supplementary Figures 1, 2; Nishizawa-Yokoi et al., 2015). In Hd1::Hd1-LUC, the first Hd1 refers to the endogenous Hd1 promoter. In Ghd7::Ghd7-LUC, the first Ghd7 refers to the endogenous Ghd7 promoter.

### Transformation for Positive and Negative Selection to Establish Gene-Targeted Lines

Both the pKOD4/Hd1::Hd1-LUC pHPTb and pKOD4/Ghd7::Ghd7-LUC pHPTb vectors were transformed into rice [*Oryza sativa L.* cultivar (cv.) Nipponbare] using *Agrobacterium*-mediated transformation (Figure 1A). When the T-DNA fragment is introduced *via* T-DNA border-associated integration by the *Agrobacterium* machinery at random positions in the rice genome and the negative selection marker is also incorporated into the genome, the transformed cells would die. However, when only the DNA fragment is inserted into the target locus through HR, the DNA sequences flanking the positive selection marker gene, *HPT*, would be incorporated into the genome, whereas the negative selection marker gene, *DT-A*, would not. As a result, transformed cells can survive as a GT line. After regeneration from the transformed calli, plants containing the GT *LUC* gene are generated. Using this *Agrobacterium* infection method for positive and negative selection, more than a thousand seeds were used to induce rice calli, and hundreds of hygromycin-resistant calli were obtained. These calli were further screened through PCR to ensure that the GT was successful. Notably, most of the hygromycin-resistant calli were obtained from RI events but not *via* the T-DNA border-associated integration and did not contain both T-DNA borders and the *DT-A* gene (Supplementary Figure 3). As a result, a single GT line designated *Ghd7::Ghd7-LUC(GT,HPTb)*, which contained the *LUC* gene fused to the *Ghd7* gene in-frame, was obtained by screening more than 500 hygromycin-resistant calli (Table 1). Then, some calli belonging to the *Ghd7::Ghd7-LUC(GT,HPTb)* line were maintained, and the hyperactive *Piggybac* transposase (*hyPBase*) expressing vector was transformed into them *via* infection with *Agrobacterium* to remove the *HPT* gene and surrounding IRs (Nishizawa-Yokoi et al., 2014). A total of 20 independent geneticin-resistant calli were obtained from which 18 lines were selected through PCR screening. We confirmed that the marker was fully removed in 15 lines through sequencing. Thus, the removal efficiency was 75% (15/20). Then, one regenerated line, designated *Ghd7::Ghd7-LUC(GT)*, was selected for further analysis (Supplementary Figure 4).

**TABLE 1:** Results of transformation experiments at National Agriculture and Food Research Organization (NARO).

		The number of seeds	Callus (g)	The number of calli	The number of hygromycin resistant calli	PCR screening		
						5′HR(+)	3′HR(+)	LUC(+)
*Hd1*	1st	160	34.58	4608	-	-	-	-
	2nd	160	13.86	2779	91	0	0	3
	3rd	320	17.667	4673	16	0	0	3
	4th	320	36.228	6524	32	0	0	n.d.
	5th	320	38.86	6599	52	0	0	n.d.
	**Total**	**1280**	**141.195**	**25183**	**191**	**0**	**0**	**6**

*Ghd7*	1st	176	30.96	5590	310	0	0	16
	2nd	208	20.351	3974	112	0	0	4
	3rd	320	30.4	6782	131	1	1	5
	**Total**	**704**	**81.711**	**16346**	**553**	**1**	**1**	**25**

After preliminary analysis, this *Ghd7::Ghd7-LUC(GT)* line exhibited lower LUC activity compared with the *Ghd7::Ghd7-LUC(GT,HPTb)* line. Thus, in this study, we used the *Ghd7::Ghd7-LUC(GT,HPTb)* line for all subsequent analyses. The *Ghd7::Ghd7-LUC(GT)* line is being subsequently crossed with Nipponbare to remove extra deleterious mutations associated with removal of the *HPT* gene.

For the *Hd1* gene, unfortunately, we were unable to obtain a GT line (Table 1). Instead, we obtained several *Hd1::Hd1-LUC* lines through RI events (Supplementary Figure 5). After preliminary analysis of these *Hd1::Hd1-LUC* lines, we selected one *Hd1::Hd1-LUC* line and confirmed that this line could be used to monitor the Hd1 gene product in real time. We designated this line *Hd1::Hd1-LUC(RI,HPTb)-1.*

### Confirmation of Gene Targeting

**FIGURE 2:**
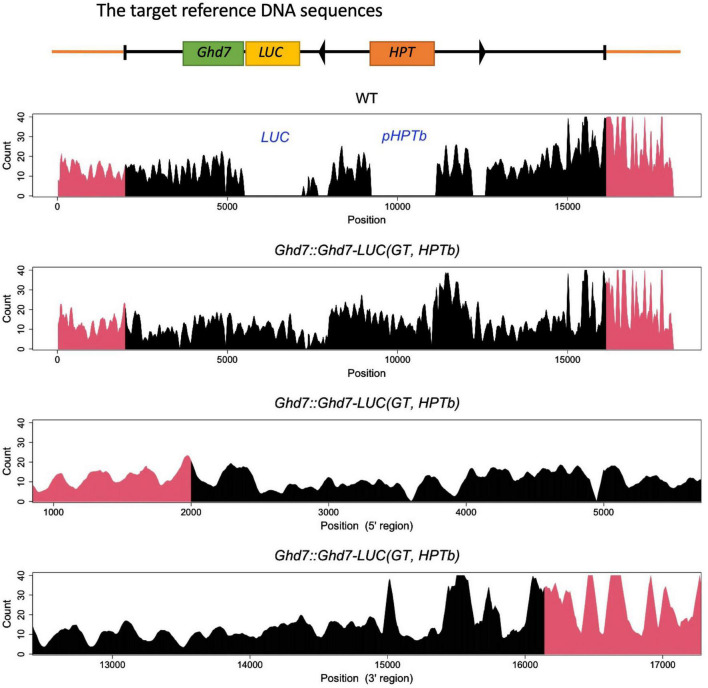
*K*-mer Analysis (*k* = 50) in WT and *Ghd7::Ghd7-LUC(GT,HPTb)* line. It was shown that *Ghd7::Ghd7-LUC(GT,HPTb)* is a valid GT line by *k*-mer analysis. The expected sequence for *Ghd7::Ghd7-LUC(GT,HPTb)* line was used as the target reference DNA sequence for *k*-mer analysis. 6 Gb illumina fastq data of both WT and *Ghd7::Ghd7-LUC(GT,HPTb)* line were analyzed to count the perfect match in tested fastq data with each *k*-mer sequence which is produced by scanning the target reference DNA sequence [*Ghd7::Ghd7-LUC(GT,HPTb)* line]. Genomic sequences outside the construct (Red region) and sequences in the construct array (Black region). The gaps in WT indicates it doesn’t have LUC, IR, and HPT. Magnified 5′ and 3′ regions where the homologous recombination must have occurred are shown with smoothing using the moving average method for intact *k*-mer values.

Three independent methods were used to confirm that the GT *Ghd7::Ghd7-LUC(GT,HPTb)* line was properly selected. For the PCR method, primer sets were designed to amplify PCR bands of the expected size (Figure 1B and Supplementary Table 1). Then, a set of scanning PCR fragments covering the entire *Ghd7::Ghd7-LUC* gene was sequenced and verified through conventional Sanger sequencing. For Southern blot hybridization experiments, electrophoresed DNA fragments were transferred to a Hybond N+ (Fisher Scientific Inc.) nylon membrane, and probes labeled with Alkphos Direct Labeling Reagents (GE Healthcare Amersham) were hybridized according to the protocol of the CDP-Star Detection Reagent (GE Healthcare Amersham) detection kit (Figure 1C). Southern blot hybridization analysis was performed using the *Ghd7* and *LUC* fragments as probes. *Hin*dIII and *Bam*HI enzymes were used to cleave genomic DNA from the *Ghd7::Ghd7-LUC(GT,HPTb)* line. Finally, for *k*-mer analysis, 6 Gb of Illumina fastq data were analyzed using the *k*-mer method (Itoh et al., 2020), and the number of perfect matches between tested fastq data and target reference DNA sequences were counted. A 50-bp sequence was used as the *k*-mer in this study. To verify the junction caused by GT, smoothing was performed using the moving average method for intact *k*-mer values (Figure 2 and Supplementary Figure 6). Abnormal hits, suggesting the improper integration of related sequences, were not detected in this *k*-mer analysis. All data indicated that *Ghd7::Ghd7-LUC(GT,HPTb)* is a valid GT line.

### Biological Evaluation of the *Ghd7::Ghd7-LUC(GT,HPTb)* Line

**FIGURE 3:**
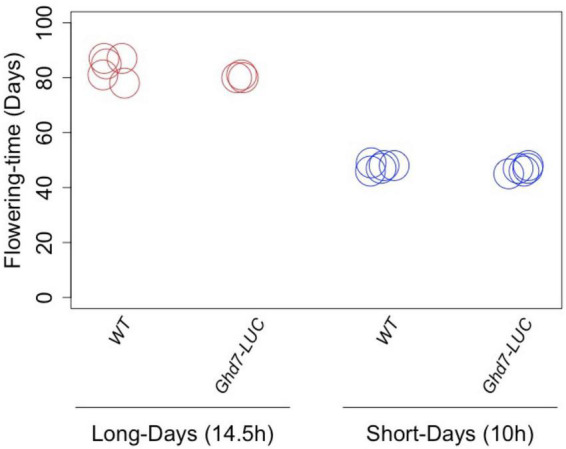
The effects of Ghd7-LUC protein on flowering time. The gene-targeted *Ghd7::Ghd7-LUC(GT,HPTb)* line exhibited normal photoperiodic flowering properly compared with the wild type. This indicates that the Ghd7-LUC protein could mimic the Ghd7 protein at least in terms of the photoperiodic control of flowering in rice. There was no significant difference in the flowering time under long-day (14.5 h of light; *p* = 0.14) and short-day (10 h of light; *p* = 0.20) conditions (*p* < 0.05; Student’s *t* test). Nipponbare is shown as WT, *Ghd7::Ghd7-LUC(GT,HPTb)* homo lines are shown as *Ghd7-LUC.*

Using plants of the T1 generation, we selected a homozygous *Ghd7::Ghd7-LUC(GT,HPTb)* plant for harvesting seeds that are homozygous for the *Ghd7::Ghd7-LUC* gene. Then, heading dates in this line were compared with those of wild-type rice plants (cv. Nipponbare) under long-day (14.5 h of light) and short-day (10 h of light) conditions (Figure 3). The results clearly indicated that the GT *Ghd7::Ghd7-LUC* line exhibited normal photoperiodic flowering compared with the wild type. This result clearly indicated that the Ghd7-LUC protein could mimic the Ghd7 protein, at least in terms of photoperiodic control of flowering in rice.

### Monitoring Using the *Ghd7-LUC* and *Hd1-LUC* Lines

In this study, two *LUC* lines, *Ghd7::Ghd7-LUC(GT,HPTb)* and *Hd1::Hd1-LUC(RI,HPTb)-1*, were used in our monitoring system.

**FIGURE 4:**
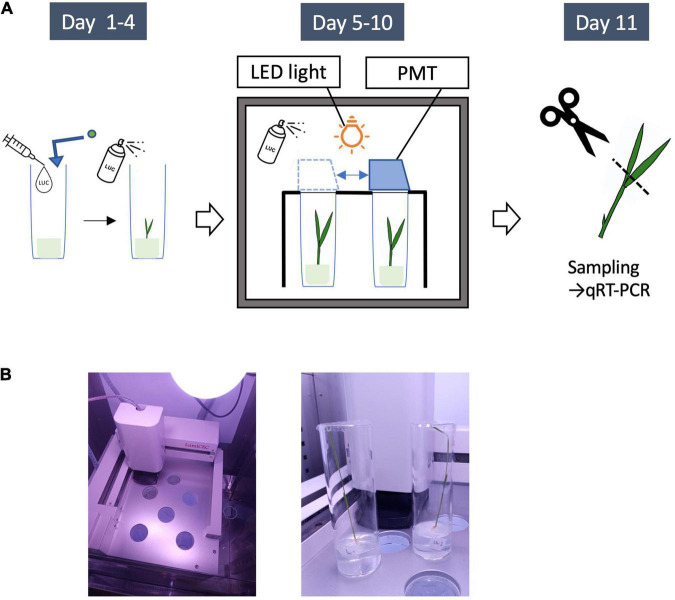
LUC Protein monitoring system. **(A)** Outline of experiments for LUC protein monitoring. Day1: Plant seeds are sown on a medium (0.2% Gellan gum + MS + 100 μl of 100 mM luciferin solution). The seedlings are germinated in the constant light incubator for 4 days. Day 4: 1 ml of LUC solution (10 mM) is sprayed. Day 5: the seedlings are put into the monitoring system. The light emissions from an entire growing rice seedling are continuously recorded by a photomultiplier tube (PMT) for 6 days. Day 9: 1 ml of LUC solution (10 mM) is sprayed again. Day 11: Leaves are sampled for quantitative RT-PCR. **(B)** Left, the monitoring unit consisting of a movable photomultiplier tube (PMT) and a LED lamp (upper right); right, the seedlings in test tubes used for this study (Day 11).

To measure LUC activity in growing rice seedlings, we developed a custom system for monitoring LUC activity using 13-cm long test tubes with a diameter of 4 cm (Churitsu Electronics Corporation, Japan). The monitoring unit consists of six test tube chambers with an automatically operated photomultiplier tube (PMT) that detects faint light in a thermostatic incubator with a controllable light-emitting diode lamp (Figure 4). With this system, light emissions from an entire growing rice seedling can be continuously recorded for around 10 days. Temporal LUC activity in six rice seedlings under the same environmental conditions can be measured at once using this system. One rice seed was sterilized and sown in each of the tubes on 50 ml Murashige and Skoog (MS) medium with 0.2% gellan gum and 100 μl of 100 mM luciferin solution, and then germinated in an incubator under constant light for 4 days. Then, after spraying with 1 ml of LUC solution (10 mM), the test tubes were placed in the PMT room, and LUC activity was measured for 3 s per minute five times. This measurement was repeated hourly at 30°C for more than 1 week. On day 9 after sowing, the seedlings were sprayed again with 1 ml of LUC solution (10 mM), and the measurements were continued. For RNA preparation, the seedlings were sampled at 1.5 h after the beginning of the light period on day 11 and immediately frozen with liquid nitrogen. After light measurements were taken, the third to fifth measured values among the five 3-s data points were averaged, and background values obtained from the negative control (non-transgenic rice seedling) were subtracted (Supplementary Figure 7). Then, the temporal LUC activity was analyzed using hourly measurements. To prevent severe contamination of the tested seeds, an antifungal agent, Plant Preservative Mixture (Plant Cell Technology Inc.), was added to the MS medium upon sowing.

## Results

### Generation of a Gene-Targeted *Ghd7::Ghd7-LUC* Line

Although abundant genetic evidence indicates the critical role of *Ghd7* in rice photoperiodic flowering, no data clearly showing genetic complementation of the *Ghd7* gene have been reported. Indeed, we attempted to transform genomic fragments including the entire coding region of *Ghd7* with a few kilobasepairs of the promoter region and a portion of the 3′ region several times, but we succeeded in only partially complementing *ghd7* deficiency (Itoh et al., 2010). Thus, we speculate that the regulatory region of the *Ghd7* gene is relatively long in the rice genome. Supporting this speculation, the upstream region of the *Ghd7* gene is rich in repeats and no genes have been annotated more than 50 kb upstream of the *Ghd7* gene in the Rice Annotation Project Database. Therefore, we attempted to construct a GT *Ghd7-LUC* line in the present study. We have demonstrated previously that the Ghd7 protein interacts with the Hd1 protein to function as a strong repressor in rice cells (Nemoto et al., 2016). Thus, we attempted to construct a complete GT *Hd1-LUC* line despite a few kilobasepairs of the promoter region being sufficient to complement *hd1* deficiency when the corresponding genomic fragment of *Hd1* is transformed (Yano et al., 2000).

We initially performed six and seven positive and negative selection experiments for *Ghd7::Ghd7-LUC* and *Hd1::Hd1-LUC*, respectively, in a laboratory at the University of Tokyo (Supplementary Table 2). In this experiment, we obtained 37 *Ghd7::Ghd7-LUC* transformed lines, including 15 lines that targeted only the 5′ promoter region but not the 3′ region. The others exhibited RI of *Ghd7::Ghd7-LUC* along with the removal of the *DT-A* genes. Meanwhile, we obtained 23 *Hd1::Hd1-LUC* transformed lines without the *DT-A* genes, but no GT lines. These lines also exhibited RI of *Hd1::Hd1-LUC* (Supplementary Table 2). Then, we performed three and five additional selection experiments for *Ghd7::Ghd7-LUC* and *Hd1::Hd1-LUC*, respectively, in a laboratory at the National Agriculture and Food Research Organization (NARO; Table 1) in which positive and negative selection for GT in rice has been successfully performed previously. We obtained one fully GT *Ghd7::Ghd7-LUC* line and 24 RI *Ghd7::Ghd7-LUC* lines. Meanwhile, six RI *Hd1::Hd1-LUC* lines, but no GT *Hd1::Hd1-LUC* lines, were obtained (Table 1). We designated the GT *Ghd7::Ghd7-LUC* line as *Ghd7::Ghd7-LUC(GT,HPTb)* and selected one RI *Hd1::Hd1-LUC* line with similar levels of LUC activity to *Ghd7::Ghd7-LUC(GT,HPTb)* upon preliminary measurement and designated it *Hd1::Hd1-LUC(RI,HPTb)-1.* These two lines were used for further analysis in this study.

### The Efficiency of Gene Targeting in This Study

Using transformation data from NARO, the efficiency of GT was estimated. In total, 191 and 553 calli were selected as hygromycin-resistant lines among 25,183, and 16,346 *Agrobacterium*-infected calli for *Hd1* and *Ghd7*, respectively. This result indicates that 0.76 and 3.4% of the calli exhibited transformation into the rice genome without integration of the *DT-A* genes for *Hd1* and *Ghd7*, respectively. Most of those transformations were considered RI insertion events, rather than events due to proper T-DNA integration. By contrast, as only one callus among the 553 resistant calli was a GT line, the RI event appears to have occurred at least 500 times more frequently than the GT event in this case. Similar results were obtained for *Hd1::Hd1-LUC*, and we were unable to produce a GT line due to the screening of only 191 hygromycin-resistant calli for *Hd1::Hd1-LUC*. However, the efficiency of hygromycin-resistant callus production was variable among experiments in this study (Table 1) and was not easily controlled. The overall efficiency based on the number of *Agrobacterium*-infected calli was 0.006% (1/16, 346) in this study (Supplementary Table 3).

### Diurnal Dynamics of the *Ghd7* and *Hd1* Proteins Under Long-Day and Short-Day Conditions

**FIGURE 5:**
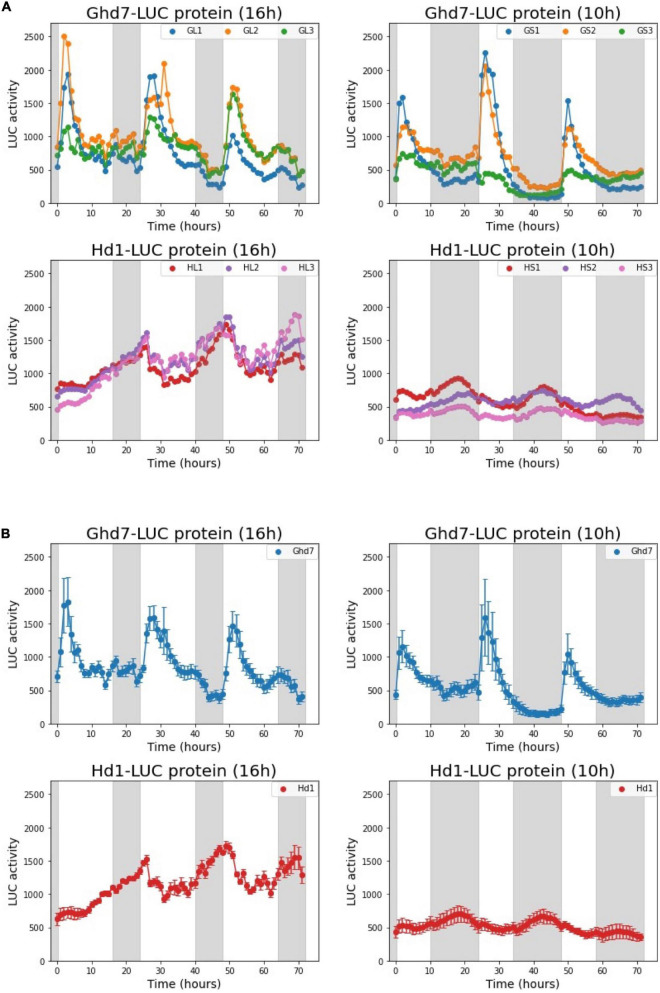
Diurnal dynamics of the Ghd7-LUC and Hd1-LUC proteins under long-day (16 h) and short-day (10 h) conditions. The diurnal patterns of Ghd7-LUC (Day 8–10) and Hd1-LUC (Day 6–8). Ghd7-LUC protein patterns in six *Ghd7::Ghd7-LUC(GT,HPTb)* homo plants under long-day (GL1,GL2,GL3) and short-day (GS1,GS2,GS3) conditions. Hd1-LUC protein patterns in six *Hd1::Hd1-LUC(RI,HPTb)-1* homo plants under long-day (HL1,HL2,HL3) and short-day (HS1,HS2,HS3) conditions. The diurnal LUC activity dynamics for each plant were recorded for consecutive 72 h. The representative data from a few independent experiments for both day-length conditions were presented. Shades represent dark periods. **(A)** The diurnal patterns of Ghd7-LUC and Hd1-LUC proteins for 72 h. **(B)** The mean and standard error of the three samples in panel **(A)** are displayed. Shades represent dark periods.

Using the *Ghd7::Ghd7-LUC(GT,HPTb)* and *Hd1::Hd1-LUC(RI,HPTb)* lines, we examined the diurnal LUC activity patterns of the Ghd7-LUC and Hd1-LUC proteins. Five-day-old seedlings were transferred to the LUC luminescence measurement device after sowing, and LUC activity was continuously monitored for 6 days under a 16-h (long-day) or 10-h (short-day) photoperiod at 30°C. Thus, with this system, we can mimic the temporal dynamics of the Ghd7 and Hd1 proteins through monitoring of LUC activity under controlled conditions (Figure 5). We found that the Ghd7-LUC protein began to accumulate rapidly after dawn and peaked at around 2 and 3 h later under short-day and long-day conditions, respectively. This result clearly indicates that the Ghd7 protein accumulated faster just after dawn under the short-day versus the long-day condition. In addition, Ghd7-LUC began to decrease after peaking more rapidly under the short-day condition than under the long-day condition. Furthermore, Ghd7-LUC began to increase slowly a few hours before dusk only under the long-day condition. After dusk, Ghd7-LUC began to decrease to its minimum level before dawn under both short-day and long-day conditions. Taken together, these results show that the level of Ghd7-LUC was clearly higher during daytime under a long-day condition than under a short-day condition. Interestingly, no significant difference was found in the peak levels of Ghd7-LUC protein between long-day and short-day conditions, suggesting that Ghd7 repressor activity may not be well represented by a snapshot of the Ghd7 protein level. Some interactions of Ghd7 protein with other factors or modifications of its structure might have contributed to these observations.

Furthermore, we found that under the long-day condition, the Hd1-LUC protein accumulated very slowly from a few hours before dusk to a few hours after dawn, reached its peak, and began to decrease rapidly until a few hours before dusk. By contrast, under the short-day condition, Hd1-LUC accumulated very slowly from a few hours before dusk to a few hours before dawn, then reached its peak, and began to decrease gradually until a few hours before dusk. Notably, the amplitudes of the Hd1-LUC peaks were clearly smaller under the short-day condition compared with the long-day condition. Interestingly, the peak and total amounts of Hd1-LUC protein were a few times higher under the long-day condition than under the short-day condition. Both the Ghd7-LUC and Hd1-LUC dynamic patterns under a long-day condition are consistent with a previous finding stating that the Ghd7 protein interacts with the Hd1 protein to create a strong repressor complex under long-day conditions. However, the data produced in this work cannot explain the activation of downstream genes by *Hd1* alone at night under short-day conditions.

### Snapshot Comparison of Ghd7-LUC and Hd1-LUC Protein Levels With mRNA Levels of Related Genes

**FIGURE 6:**
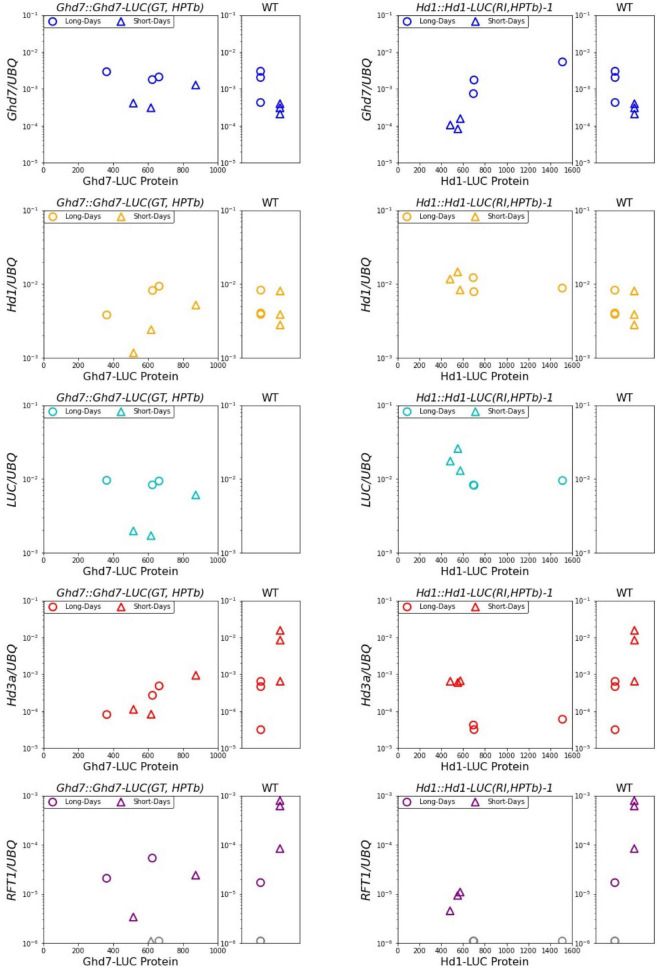
Ghd7-LUC and Hd1-LUC Proteins and related mRNA pattern. Scatter plot of LUC proteins activity and mRNA transcription level under long-days and short-days conditions. The amount of LUC proteins indicates monitored values of LUC activity at 1 h after light-on on the day 11. The corresponding amount of mRNA of related genes were shown. The leaves were sampled at 1.5 h after light-on. *LUC* mRNA was not detected in WT. The transcript levels of *RFT1* were too low to be detected in some lines (showed as gray). Primer and probe sequences are listed in Supplementary Table 1.

As a first step to elucidate the regulatory mechanisms of downstream gene expression, both the mRNA levels of related genes and Ghd7-LUC and Hd1-LUC protein levels were simultaneously examined. After 6 days of LUC activity monitoring, the leaves of 10-day-old seedlings were sampled in the morning (1.5 h after sunrise) under both long-day and short-day conditions (Figure 4). Then, we measured the mRNA levels of *Ghd7, Hd1, LUC, Hd3a*, *RFT1*, and *Ehd1* using quantitative reverse-transcription PCR (Figure 6). Comparing Ghd7-LUC and Hd1-LUC protein levels with mRNA expression of flowering-time genes in the *Ghd7::Ghd7-LUC(GT,HPTb)* and *Hd1::Hd1-LUC(RI,HPTb)-1* lines, respectively, we found that the transcript level of *Ghd7* mRNA was about 5–6 times higher under the long-day condition than under the short-day condition, but no significant difference in the activity of Ghd7-LUC protein was observed (Figures 5, 6). This result suggests that the *Ghd7* mRNA level may not reflect the amount of Ghd7 protein at a given moment, perhaps due to a lag in the translation process or mechanisms controlling Ghd7 protein stability. A similar result was obtained for the relationship between Hd1-LUC activity and *Hd1-LUC* mRNA in the *Hd1::Hd1-LUC(RI,HPTb)* line.

Unexpectedly, in the *Ghd7::Ghd7-LUC(GT,HPTb)* line, the levels of *Hd3a* and *RFT1* mRNA were low in the morning under both short-day and long-day conditions. This response of *Hd3a* and *RFT1* mRNA is distinct from that in wild-type plants. This result means that Ghd7-LUC protein may have stronger repressor activity than the endogenous Ghd7 protein at least on this tested developmental stage. Thus, more careful evaluation to reveal the relationship between downstream gene expression and Ghd7-LUC levels in the *Ghd7::Ghd7-LUC(GT,HPTb)* line using seedlings of different stages and timings is essential. In the *Hd1::Hd1-LUC(GT,HPTb)* line, the amount of Hd1 protein may be weakly negatively correlated with the *Hd3a* mRNA level in the morning (Figure 6).

## Discussion

### Efficiency of Gene Targeting

The efficiency of GT in plants was first reported to be 0.005–0.042% based on the ratio of the number of antibiotic-resistant clones obtained from GT to the number of antibiotic-resistant clones obtained through transformation using protoplasts (Paszkowski et al., 1988). The ratio of the number of transformed calli to that of calli with NPTII genes repaired through GT was approximately 0.02% when the repair construct was transformed *via Agrobacterium* infection into tobacco protoplasts (Offringa et al., 1990). Using positive and negative selection, the ratio of PCR-positive clones was approximately 1% (Terada et al., 2002). Thus, positive and negative selection is an effective method for improving the success rate of GT experiments. In this study, we performed extensive positive and negative selection but obtained only one line, resulting in an estimated GT efficiency for *Ghd7* of approximately 0.2%, or 1/553 PCR-positive clones. Based on infected callus numbers, the success rate was estimated as 0.006% (1/16,346). In addition, no GT line was obtained for *Hd1*. Thus, our trial results can be considered within the typical range for GT experiments in plants, although the rate of RI with removal of the *DT-A* genes in this study may be higher than in previous reports. A better method than positive and negative selection needs to be developed for use in plants. A few years ago, a GT method based on targeted DSB events using CRISPR/Cas9 was reported (Miki et al., 2018). In that study, transformation of both the Cas9 gene and sgRNA expression unit occurred independently, but the apparent efficiency of obtaining GT lines was clearly higher than in previous reports. Thus, the efficiency of GT experiments in plants can be expected to improve greatly over the next few years since there is a report indicating an improvement of GT using CRISPR/Cas9 induced DSBs in various plants (Nishizawa-Yokoi et al., 2020). Along with the DSBs induced by CRISPR/Cas9, the use of *DT-A* negative selection to reduce RI may be key to more reliable GT in plants.

### Development of a Monitoring System

In this study, we developed a real-time monitoring system for two key flowering-timing compounds−the gene products of *Ghd7* and *Hd1*−in rice. We previously monitored the diurnal dynamics of the LUC-fused *OsLHY* gene under control of the CaMV 35S promoter. In that study, we successfully demonstrated that light conditions clearly affected OsLHY-LUC protein stability (Ogiso et al., 2010). Using these systems with the *Ghd7::Ghd7-LUC* and *Hd1::Hd1-LUC* lines, we should be able to monitor protein stability under controlled environmental conditions in addition to achieving translational control of these genes. Similarly, using *LUC* as a reporter gene, tissue-specific rhythmic expression under the control of circadian clocks has been reported using split N-half and C-half *LUC* genes (Endo et al., 2014). In addition, auxin-dependent control of protein degradation was clearly observed using *LUC* reporter genes (Salmon et al., 2007). These examples guarantee the usefulness of the LUC-fusion monitoring system in plants. In this study, because GT integration of the *LUC* gene with the *Ghd7* gene in an in-frame manner was established in the *Ghd7::Ghd7-LUC(GT,HPTb)* line and the *Ghd7-LUC* gene product could confer the photoperiodic flowering trait, we are certain that real-time monitoring of the Ghd7 protein can be successfully conducted under various environmental conditions and with diverse genetic backgrounds. In the *Hd1::Hd1-LUC(RI,HPTb)* line, because a sufficient length of the promoter region of the *Hd1* gene was integrated into the rice genome and reasonable LUC activity was observed, we expect that this line will be useful for temporal monitoring of the *Hd1* gene product. We are now introducing this RI *Hd1::Hd1-LUC* gene into *hd1*-defective lines to confirm whether the *Hd1-LUC* allele can mimic *Hd1* function.

### Diurnal Dynamics of the *Ghd7* and *Hd1* Proteins Differ From Those of *Ghd7* and *Hd1* mRNA

We previously measured the mRNA dynamics of *Hd1* and *Ghd7* using the cv. Hoshinoyume background with nearly isogenic lines under 14.5-h long-day and 10-h short-day conditions (Nemoto et al., 2016). Although we should be cautious about describing the differences between the dynamics of *Hd1* and *Ghd7* mRNA and gene products, we can compare mRNA dynamics with the LUC data obtained in this study.

For *Ghd7* mRNA, a rapid increase after dawn was clearly observed under both long-day and short-day conditions. *Ghd7* mRNA levels were around 10^–4^ and 10^–2^ (after normalized by copy numbers of an endogenous *UBQ* mRNA) at 1 h before and 1 h after light-on under the long-day condition (14.5 h), respectively, in the cv. Hoshinoyume background. Meanwhile, these levels were less than 10^–4^ and less than 10^–2^ under the short-day condition (10 h). After those increase in the morning, *Ghd7* mRNA levels decreased slowly and then increased slightly just before dusk, followed by a decrease until dawn under the long-day condition, whereas they decreased slowly until dawn under the short-day condition. These patterns were similar to those of Ghd7-LUC activity, except the speed of accumulation of the Ghd7-LUC protein in the morning. The accumulation of Ghd7-LUC was more rapid under the short-day condition than under the long-day condition. This difference in the increase of Ghd7-LUC just after dawn under distinct photoperiods may be associated with the repressor activity of Ghd7 or the formation of a Ghd7 repressor complex with other factors such as the Hd1 protein. As Ghd7 repressor complex activity was apparent under the long-day condition, but not under the short-day condition, the slow increase in Ghd7-LUC after dawn under the long-day conditions may be related to the formation of the repressor complex.

For *Hd1* mRNA, typical sine curve-like dynamics were observed under the short-day condition after log-transformation and calibration. The trough of *Hd1* mRNA was slightly above 10^–3^ around noon, whereas the peak *Hd1* mRNA level was 10^–1^ under the short-day condition. Under the long-day condition, the amplitude between Hd1 mRNA peaks and troughs was larger during daytime, but the dynamics of mRNA levels at night were unclear. The trough level of *Hd1* mRNA was around 10^–3^ and the peak value was around 10^–1^ under the long-day condition. The rapid decrease in *Hd1* mRNA after dawn was clearer under the long-day condition than under the short-day condition. In this work we found that Hd1-LUC dynamics were quite distinct from these mRNA dynamics. Compared with the reported *Hd1* mRNA and Hd1-LUC activity levels, the rapid decrease in *Hd1* mRNA just after dawn may reflect the rapid loss of Hd1-LUC activity under a long-day condition, whereas the increase in Hd1-LUC at night under a long-day condition might have been due to the relatively constant level of *Hd1* mRNA at night. However, the low Hd1-LUC activity under the short-day condition is not easily explained. The translation or stability of Hd1-LUC may be differently regulated under short-day conditions compared with long-day conditions. After seed propagation of these reporter lines, mRNA dynamics and the dynamics of LUC-fused proteins will be examined using the same plants under distinct photoperiod conditions. Although we monitored LUC activity hourly in this work, it is possible to monitor LUC activity more often. Thus, real-time monitoring with a high temporal resolution will be attempted in the near future.

### Snapshot Comparison Between Ghd7-LUC/Hd1-LUC Activity Levels and Related mRNA Levels

At the end of the LUC measurement period, in this work, we sampled RNA from the same seedlings to compare mRNA levels of related genes and the LUC activity level simultaneously in a single plant. We found that the *Ghd7* mRNA level was 5–6 times higher in the morning under a long-day condition (16 h) than under a short-day condition (10 h), but observed no significant difference in Ghd7-LUC activity in *Ghd7::Ghd7-LUC(GT,HPTb)* (Figure 6). This result may reflect a time lag related to the translation process from mRNA to protein, indicating the importance of monitoring gene products to reveal the entire molecular mechanism. For *Ehd1*, a downstream gene of *Ghd7*, mRNA level was 4–5 times higher under the short-day condition than the long-day condition in *Ghd7::Ghd7-LUC(GT,HPTb)*. *Ehd1* transcript levels and Ghd7-LUC activities are negatively correlated weakly in the morning snap-shot samples, implying that Ghd7-LUC protein may repress *Ehd1* transcription directly. Similarly, *Ehd1* transcript levels and Hd1-LUC activities are negatively correlated in the morning snap-shot samples, implying that Hd1-LUC protein may repress *Ehd1* transcription directly. Unexpectedly, in contrast to data obtained using wild-type plants, *Hd3a* and *RFT1* mRNA were clearly repressed at this seedling stage under both short-day and long-day conditions in *Ghd7::Ghd7-LUC(GT,HPTb)* (Figure 6). Thus, the significance of Ghd7-LUC activity in controlling downstream genes cannot be assessed based on this work, although Ghd7-LUC proteins may control *Ehd1* gene expression. By contrast, clear differences were observed in *Hd3a* and *RFT1* mRNA levels between short-day and long-day conditions in *Hd1::Hd1-LUC(RI,HPTb)*, and a clear association was found between the *Hd3a* mRNA (and *RFT1* mRNA) level and Hd1-LUC activity, indicating that the Hd1-LUC protein level may reflect transcriptional activity for *Hd3a* and *RFT1* genes during this snapshot observation. It is known that the *Hd1* gene can activate the *Hd3a* and *RFT1* genes at night under short-day conditions (Nemoto et al., 2016). Thus, various samples collected at distinct times under short-day conditions should be examined in the future.

### Possibility of a Hd1-Ghd7 Protein Complex

Hd1 and Ghd7 proteins form a complex together during the day under long-day conditions, which suppresses downstream genes such as *Ehd1*, *Hd3a*, and *RFT1* (Nemoto et al., 2016; Goretti et al., 2017; Zhang et al., 2017). In this study, we found that Ghd7-LUC activity during daytime after its peak was higher under a long-day condition than under a short-day condition, indicating that the Ghd7 protein is present at high levels for a longer period during daytime under long-day conditions. By contrast, no significant difference was found in the peak level of Ghd7 protein between long-day and short-day conditions. This finding suggests that Ghd7 repressor activity may be determined by not only the protein level but also interactions with other factors or modification of the Ghd7 protein. The higher level of Hd1-LUC protein under a long-day condition was consistent with repressor complex formation between Ghd7 and Hd1 under long-day conditions. We previously demonstrated that a 30-min difference in day length could affect the control of downstream genes such as *Hd3a* and *RFT1* by both *Ghd7* and *Hd1* (Itoh et al., 2010). Thus, using the monitoring system described in this study, detailed analysis of photoperiodic control of downstream genes under distinct day lengths will be performed in future works.

## Conclusion

We developed a real-time monitoring system for both Ghd7 and Hd1 gene products involved in photoperiodic flowering in rice, using GT with the LUC reporter gene fused to the *Ghd7* gene in frame and using RI with the LUC reporter gene fused to the *Hd1* gene in frame to reveal the detailed molecular mechanisms of photoperiodic flowering at the protein level.

## Data Availability Statement

The datasets presented in this study can be found in online repositories. The names of the repository/repositories and accession number(s) can be found in the article/Supplementary Material.

## Author Contributions

TI organized all this work and revised this manuscript. KK and NO developed the LUC lines. YO, HI, AN-Y, and ST supported all the experiments of the entire positive and negative selection. HY confirmed the lines and developed the monitoring system, and wrote the first draft of this manuscript. The others were also involved in the revision process.

## Conflict of Interest

The authors declare that the research was conducted in the absence of any commercial or financial relationships that could be construed as a potential conflict of interest.

## Publisher’s Note

All claims expressed in this article are solely those of the authors and do not necessarily represent those of their affiliated organizations, or those of the publisher, the editors and the reviewers. Any product that may be evaluated in this article, or claim that may be made by its manufacturer, is not guaranteed or endorsed by the publisher.

## Supplementary Material

Supplementary Figure 1 Gene-targeting construct for *Ghd7* gene. Structure of *Ghd7::Ghd7-LUC pHPTb* (the region sandwiched between RB and LB of a binary vector) and sequence of junctions between fragments. Each fragment was named F1, F2, F3, F4, and F5. Primers used to extract fragments from the genome are listed in Supplementary Table 1.

Supplementary Figure 2 Gene-targeting construct for *Hd1* gene. Structure of *Hd1::Hd1-LUC pHPTb* (the region sandwiched between RB and LB of a binary vector) and sequence of junctions between fragments. Each fragment was named F1, F2, F3, F4, and F5. Primers used to extract fragments from the genome are listed in Supplementary Table 1.

Supplementary Figure 3 *Ghd7::Ghd7-LUC(GT,HPTb)* line screening. In positive and negative selection, a callus (#64) that was positive in PCR for 5′HR, 3′HR, and GT long (see above) was selected. The regenerated shoots were hydroponically cultivated for several days, and 25 well-developed individuals from hygromycin resistant calli were potted. All individuals were tested for PCR with primer sets (5′HR, 3′HR, GT long) and confirmed to be positive. 5′HR PCR fragments were sequenced full length for 5 individuals. The black bars with triangles at the ends show the PCR primer sites. The regions indicated by the gray arrows were sequenced. Primers are listed in Supplementary Table 1.

Supplementary Figure 4 Confirmation of removal of *PiggyBac* transposon. A gene-targeted callus (#64) was divided into four plates (newly numbered #4, #7, #25, and #30) and propagated to infect the *hyPBase* construct. PCR was performed to confirm the removal of *HPT* gene in T0 generation *Ghd7::Ghd7-LUC (GT)* lines. The black bar with triangles indicate the PCR site. The regions indicated by the gray arrows were sequenced. The primer sequences are listed in Supplementary Table1.

Supplementary Figure 5 Screening of *Hd1::Hd1-LUC(RI,HPTb)-1* line. The left column name shows callus individuals, and the upper row shows the PCR range. “+” means “detected,” meanwhile “-” means “not-detected.” In 8 regenerated lines, PCR screening was performed. After preliminary monitoring of LUC activity, a line termed “AZ” was selected as *Hd1::Hd1-LUC(RI,HPTb)-1* for this study. The black bars with triangles indicate the PCR primer sites. The primer sequences are listed in Supplementary Table 1.

Supplementary Figure 6 *K*-mer analysis in WT and *Ghd7::Ghd7-LUC(GT)* line. Abnormal hits were not detected in this *k*-mer analysis. This result indicated that *Ghd7::Ghd7-LUC(GT)* is a valid GT line with the removal of *HPT* gene. **(A)** Schematic presentation of *Ghd7::Ghd7-LUC(GT,HPTb)* construct (Figure 1). **(B)** *K*-mer analysis (*k* = 50). The expected sequence for *Ghd7::Ghd7-LUC(GT,HPTb)* line was used as the target reference DNA sequence for *k*-mer analysis. 6 Gb illumina fastq data [WT and *Ghd7::Ghd7-LUC(GT)* line] were analyzed to count the perfect match in tested fastq data with each *k*-mer sequence which is produced by scanning the target reference DNA sequences [*Ghd7::Ghd7-LUC(GT,HPTb)* line]. Genomic sequences outside the construct (Red region) and sequences in the construct array (Black region). The gaps in WT indicate that the line doesn’t have LUC, IR, and pHPTb. The gap in *Ghd7::Ghd7-LUC(GT)* indicate that pHPTb was successfully removed in this line. Magnified 5′ and 3′ regions where the homologous recombination must have occurred are shown with smoothing using the moving average method for intact *k*-mer values.

Supplementary Figure 7 Ghd7-LUC and Hd1-LUC protein patterns for 6 days. The diurnal patterns of Ghd7-LUC and Hd1-LUC proteins on day 5–10 after sowing. Shades represent dark periods. Seedlings 4 days after sowing were transferred to the LUC luminescence monitoring instrument. LUC activity was continuously monitored under 16 h (long-day) or 10 h (short-day) photoperiods. After 6-days monitoring of LUC activity, the leaves of the seedlings were sampled in the morning (1.5 h after sunrise). Black arrows indicate the points when luciferin was added. After light measurements were taken, the third to fifth measured values among the five 3-s data points were averaged, and background values obtained from the non-transgenic rice seedling (WT) were subtracted. The background itself is shown as WT. Ghd7-LUC protein patterns in *Ghd7::Ghd7-LUC(GT,HPTb)* homo lines under long-day (GL1,GL2,GL3) and short-day (GS1,GS2,GS3) conditions. Hd1-LUC protein patterns in *Hd1::Hd1-LUC(RI,HPTb)-1* homo lines under long-day (HL1,HL2,HL3) and short-day (HS1,HS2,HS3) conditions.
