# Supplementary figures and images for: Real-Time Monitoring of Key Gene Products Involved in Rice Photoperiodic Flowering
Source: Front Plant Sci. 2021 Dec 15;12:766450. doi: 10.3389/fpls.2021.766450 (PMC8715009; doi:10.3389/fpls.2021.766450)

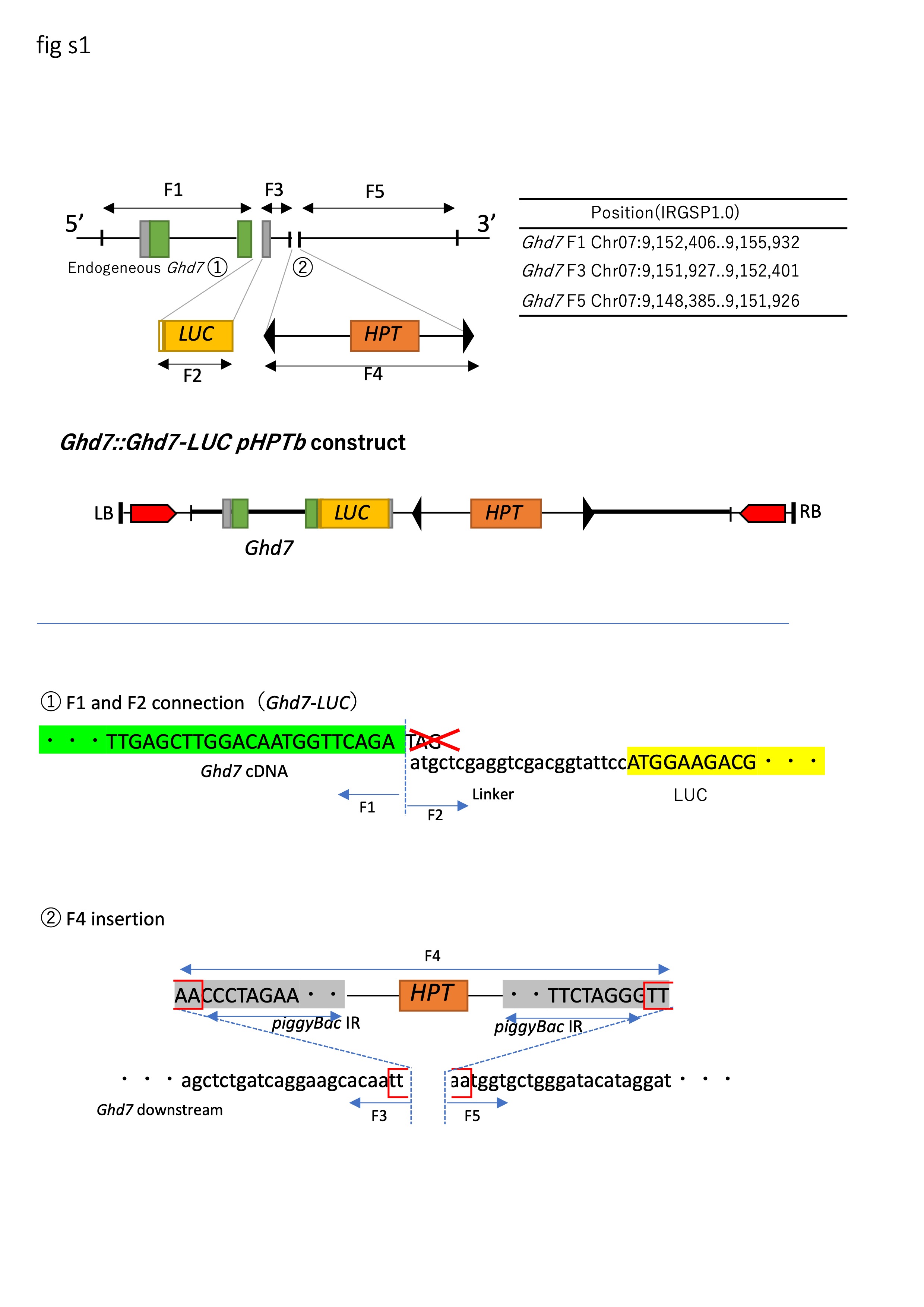

Supplement: Supplementary Figure 1 — Gene-targeting construct for Ghd7 gene. Structure of Ghd7::Ghd7-LUC pHPTb (the region sandwiched between RB and LB of a binary vector) and sequence of junctions between fragments. Each fragment was named F1, F2, F3, F4, and F5. Primers used to extract fragments from the genome are listed in Supplementary Table 1. [file Image_1.JPEG]

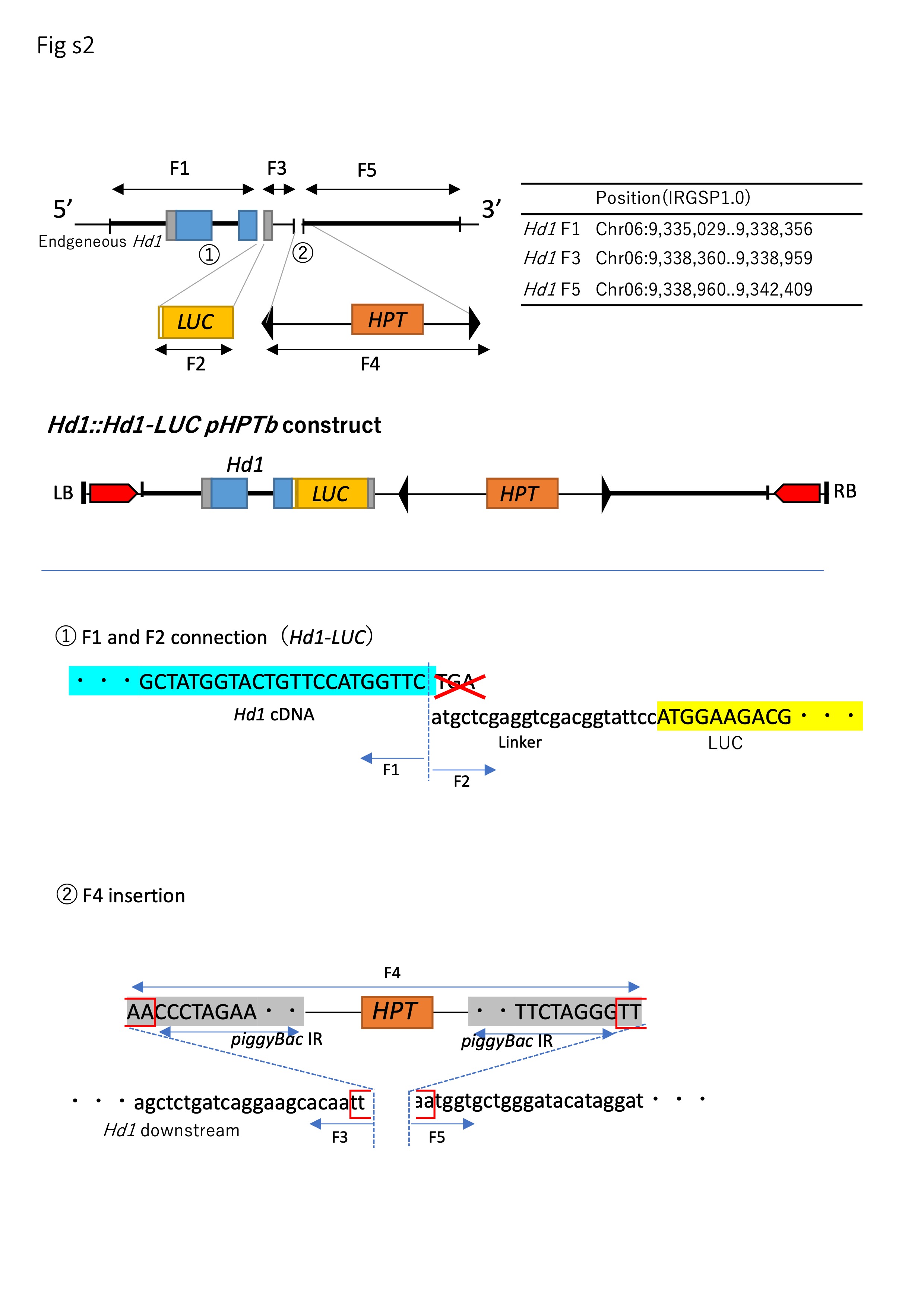

Supplement: Supplementary Figure 2 — Gene-targeting construct for Hd1 gene. Structure of Hd1::Hd1-LUC pHPTb (the region sandwiched between RB and LB of a binary vector) and sequence of junctions between fragments. Each fragment was named F1, F2, F3, F4, and F5. Primers used to extract fragments from the genome are listed in Supplementary Table 1. [file Image_2.JPEG]

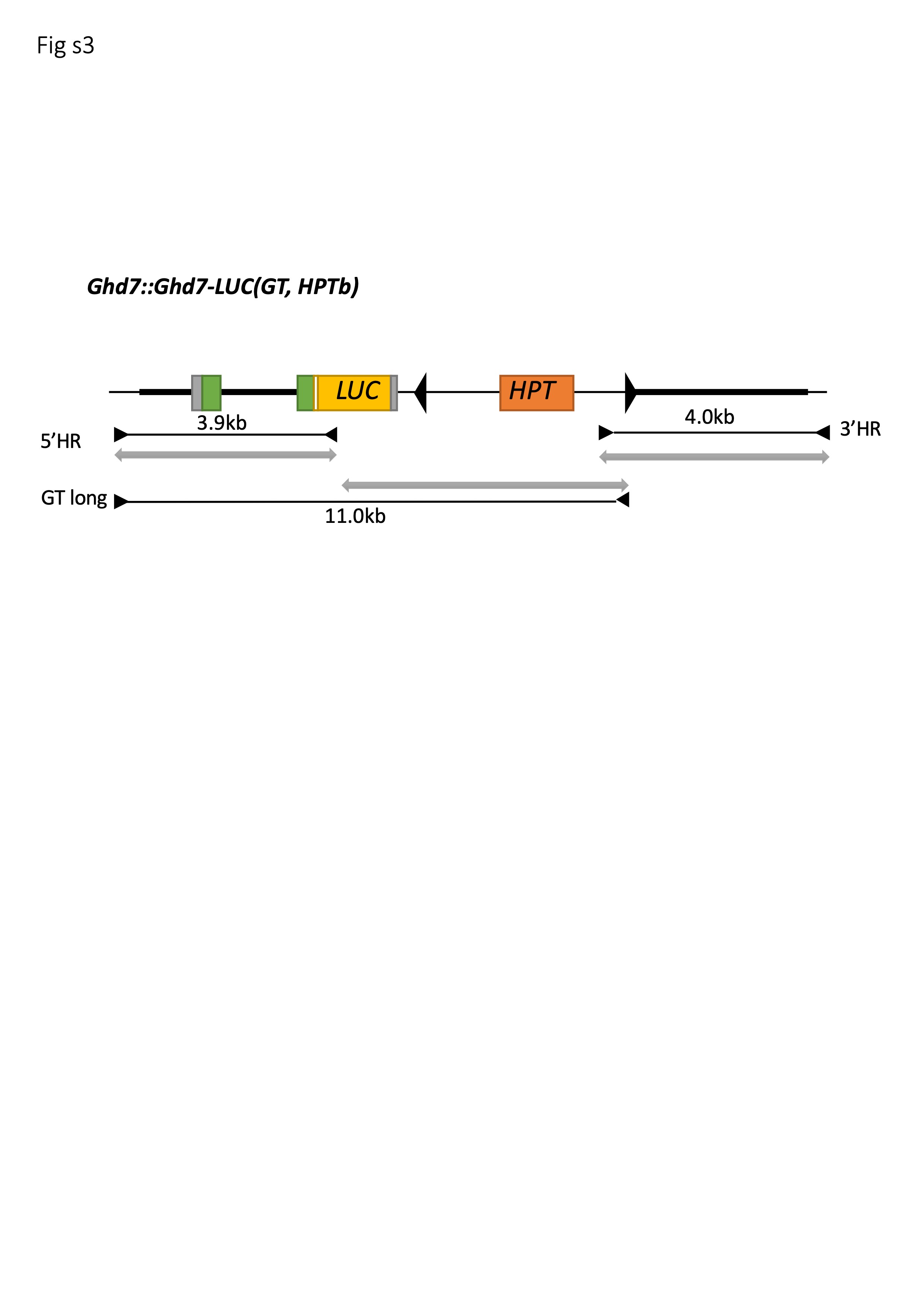

Supplement: Supplementary Figure 3 — Ghd7::Ghd7-LUC(GT,HPTb) line screening. In positive and negative selection, a callus (#64) that was positive in PCR for 5′HR, 3′HR, and GT long (see above) was selected. The regenerated shoots were hydroponically cultivated for several days, and 25 well-developed individuals from hygromycin resistant calli were potted. All individuals were tested for PCR with primer sets (5′HR, 3′HR, GT long) and confirmed to be positive. 5′HR PCR fragments were sequenced full length for 5 individuals. The black bars with triangles at the ends show the PCR primer sites. The regions indicated by the gray arrows were sequenced. Primers are listed in Supplementary Table 1. [file Image_3.JPEG]

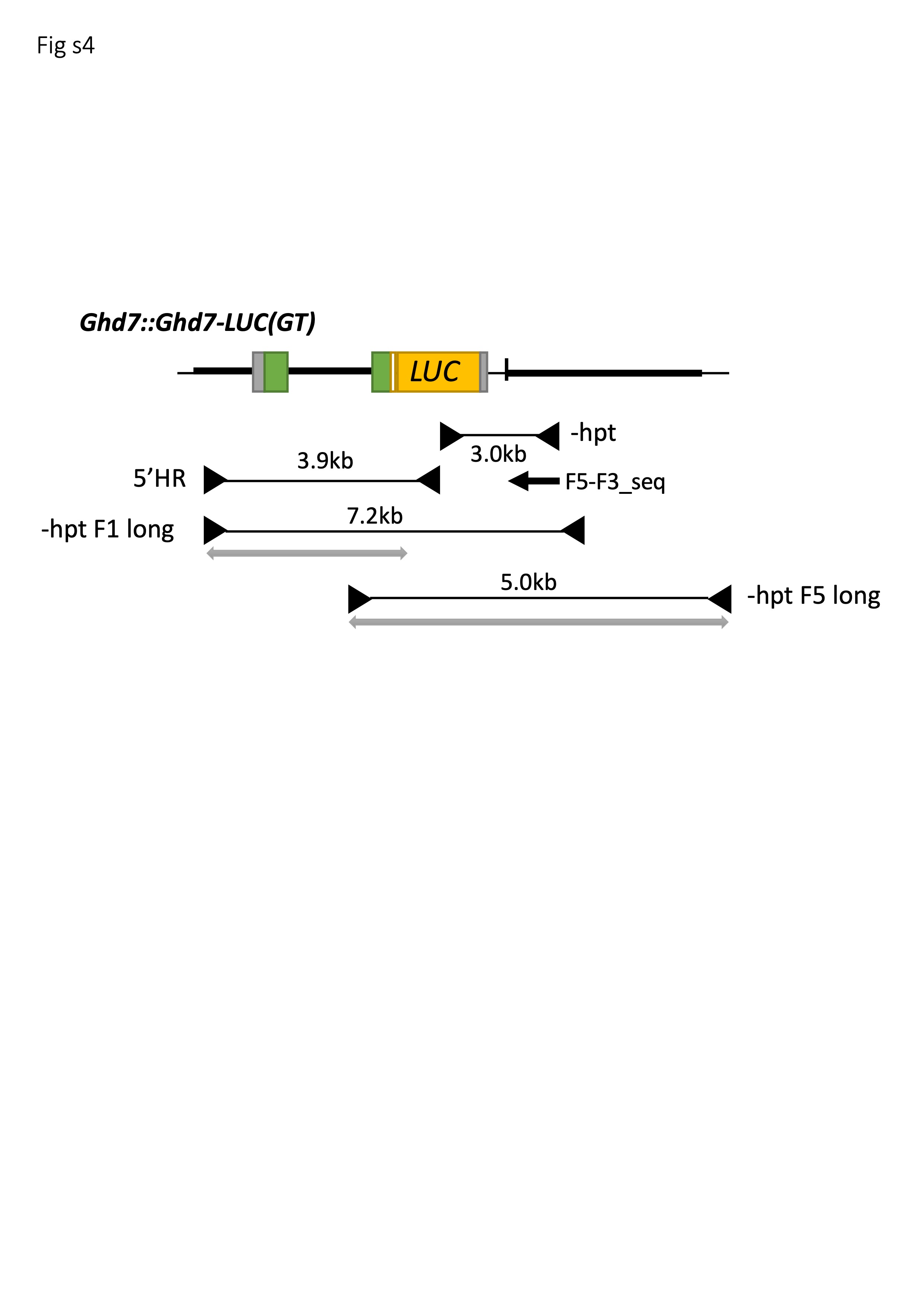

Supplement: Supplementary Figure 4 — Confirmation of removal of PiggyBac transposon. A gene-targeted callus (#64) was divided into four plates (newly numbered #4, #7, #25, and #30) and propagated to infect the hyPBase construct. PCR was performed to confirm the removal of HPT gene in T0 generation Ghd7::Ghd7-LUC (GT) lines. The black bar with triangles indicate the PCR site. The regions indicated by the gray arrows were sequenced. The primer sequences are listed in Supplementary Table1. [file Image_4.JPEG]

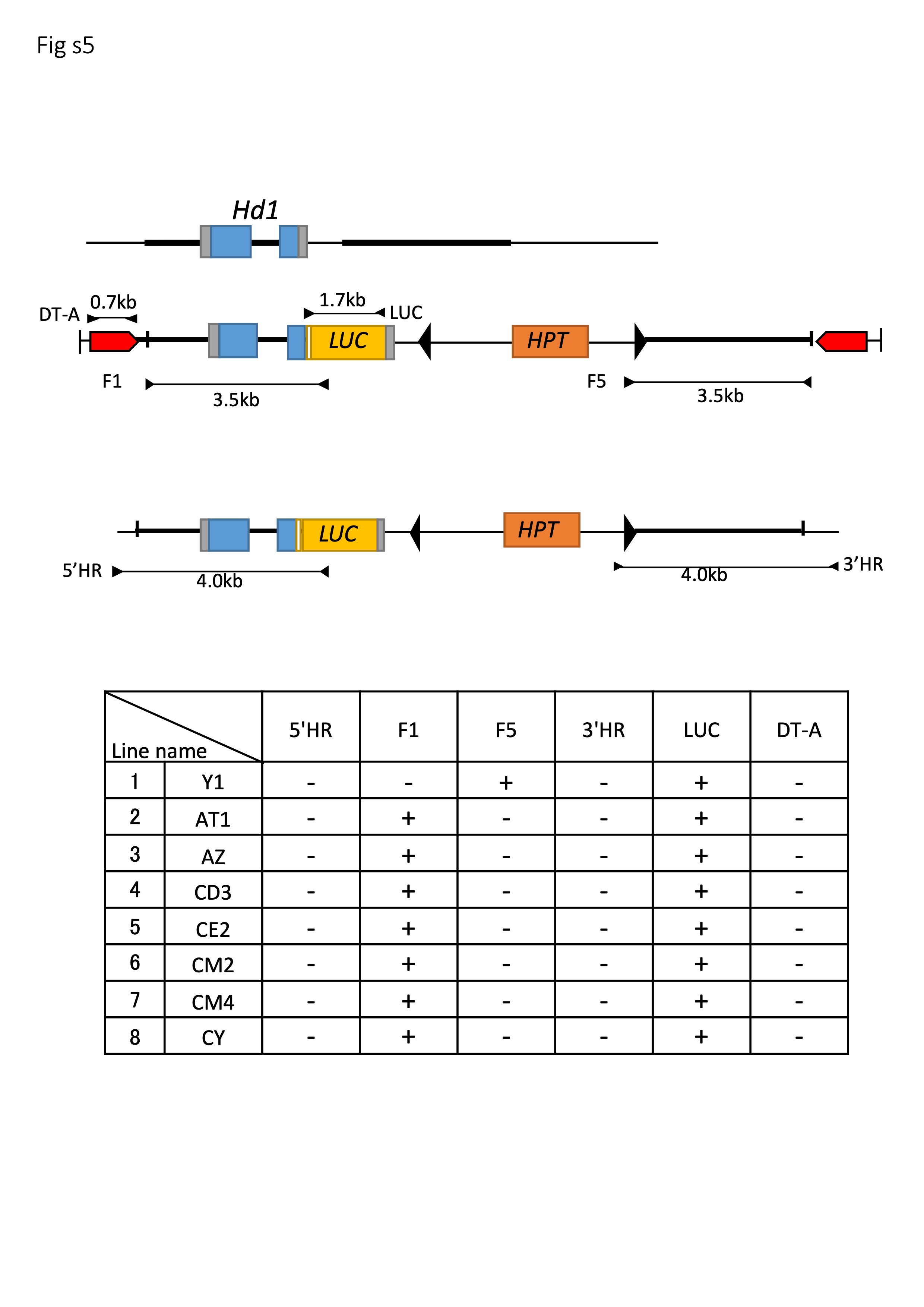

Supplement: Supplementary Figure 5 — Screening of Hd1::Hd1-LUC(RI,HPTb)-1 line. The left column name shows callus individuals, and the upper row shows the PCR range. “+” means “detected,” meanwhile “-” means “not-detected.” In 8 regenerated lines, PCR screening was performed. After preliminary monitoring of LUC activity, a line termed “AZ” was selected as Hd1::Hd1-LUC(RI,HPTb)-1 for this study. The black bars with triangles indicate the PCR primer sites. The primer sequences are listed in Supplementary Table 1. [file Image_5.JPEG]

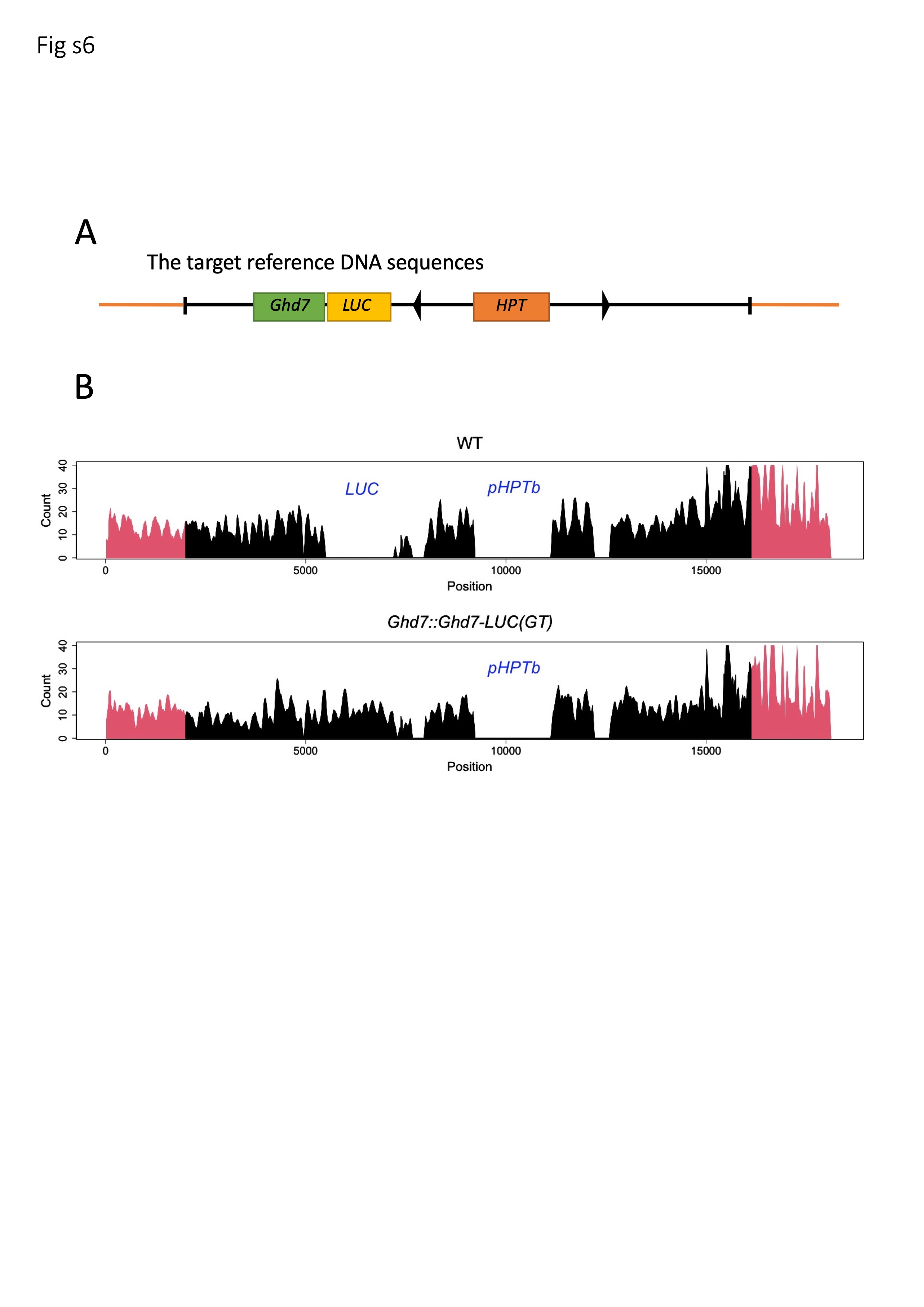

Supplement: Supplementary Figure 6 — K-mer analysis in WT and Ghd7::Ghd7-LUC(GT) line. Abnormal hits were not detected in this k-mer analysis. This result indicated that Ghd7::Ghd7-LUC(GT) is a valid GT line with the removal of HPT gene. (A) Schematic presentation of Ghd7::Ghd7-LUC(GT,HPTb) construct (Figure 1). (B) K-mer analysis (k = 50). The expected sequence for Ghd7::Ghd7-LUC(GT,HPTb) line was used as the target reference DNA sequence for k-mer analysis. 6 Gb illumina fastq data [WT and Ghd7::Ghd7-LUC(GT) line] were analyzed to count the perfect match in tested fastq data with each k-mer sequence which is produced by scanning the target reference DNA sequences [Ghd7::Ghd7-LUC(GT,HPTb) line]. Genomic sequences outside the construct (Red region) and sequences in the construct array (Black region). The gaps in WT indicate that the line doesn’t have LUC, IR, and pHPTb. The gap in Ghd7::Ghd7-LUC(GT) indicate that pHPTb was successfully removed in this line. Magnified 5′ and 3′ regions where the homologous recombination must have occurred are shown with smoothing using the moving average method for intact k-mer values. [file Image_6.JPEG]

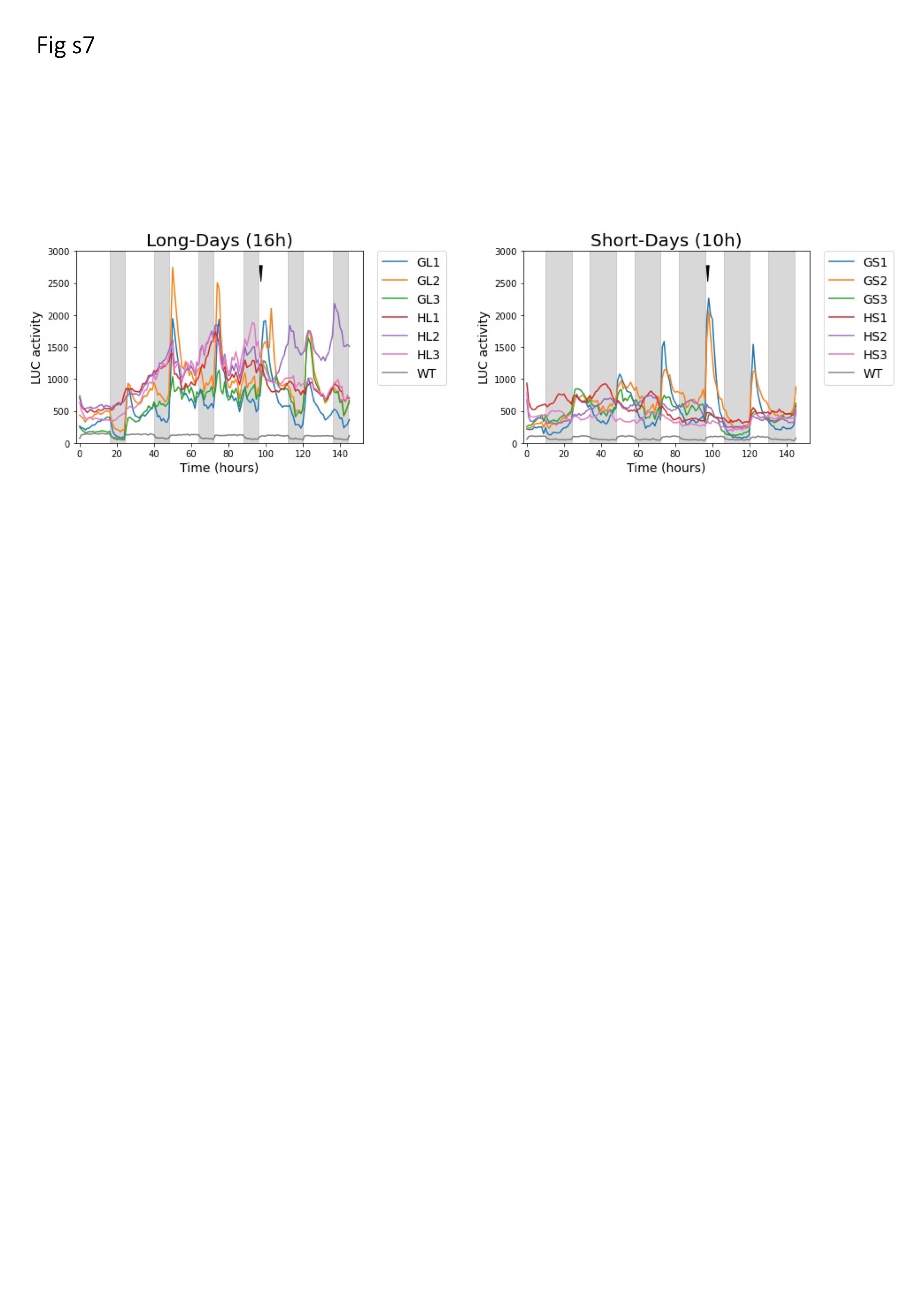

Supplement: Supplementary Figure 7 — Ghd7-LUC and Hd1-LUC protein patterns for 6 days. The diurnal patterns of Ghd7-LUC and Hd1-LUC proteins on day 5–10 after sowing. Shades represent dark periods. Seedlings 4 days after sowing were transferred to the LUC luminescence monitoring instrument. LUC activity was continuously monitored under 16 h (long-day) or 10 h (short-day) photoperiods. After 6-days monitoring of LUC activity, the leaves of the seedlings were sampled in the morning (1.5 h after sunrise). Black arrows indicate the points when luciferin was added. After light measurements were taken, the third to fifth measured values among the five 3-s data points were averaged, and background values obtained from the non-transgenic rice seedling (WT) were subtracted. The background itself is shown as WT. Ghd7-LUC protein patterns in Ghd7::Ghd7-LUC(GT,HPTb) homo lines under long-day (GL1,GL2,GL3) and short-day (GS1,GS2,GS3) conditions. Hd1-LUC protein patterns in Hd1::Hd1-LUC(RI,HPTb)-1 homo lines under long-day (HL1,HL2,HL3) and short-day (HS1,HS2,HS3) conditions. [file Image_7.JPEG]
